# Pelvic floor rehabilitation to improve functional outcome and quality of life after surgery for rectal cancer: study protocol for a randomized controlled trial (FORCE trial)

**DOI:** 10.1186/s13063-019-4043-7

**Published:** 2020-01-28

**Authors:** A.J. Kalkdijk-Dijkstra, J.A.G. van der Heijden, H.L. van Westreenen, P.M.A. Broens, M. Trzpis, J.P.E.N. Pierie, B.R. Klarenbeek, FORCE Trial Group, M. W. J. Stommel, J. H. W. de Wilt, A. J. A. Bremers, C. Rosman, P. R. de Reuver, S. A. W. Bouwense, B. M. van der Kolk, L. M. Garms, K. Meerten-van den Belt, M. R. M. Olde Hartman-Hofsté, J. W. M. Peters, L. Olsder, I. Huizing, E. J. B. Furnée, K. Havenga, P. H. J. Hemmer, B. van Etten, A. Koop, L. van der Heide, D. Kamphuis, S. A. Koopal, C. Hoff, H. Eker, H. H. M. Junte, I. J. H. Schoenaker, S. Quaedackers, M. J. Bos, H. Gardien, T. C. van Sprundel, P. D. de Vries, J. F. Ashruf, L. Geurts, I. Nielen, J. Pfeil, M. van Ark, S. W. Polle, B. Hansson, F. Polat, H. de Vries, E. ten Berge-Groen, A. K. Talsma, R. Bosker, E. Veurink, M. Papa, A. J. G. Maaskant-Braat, F. J. C. van den Broek, W. K. G. Leclercq, G. D. Slooter, F. Caers, M. Boeijen, R. van den Broek, K. van Schaik, D. K. Wasowicz-Kemps, B. S. Langenhoff, M. J. van den Bogaard, J. van der Sluis, D. Arisz, S. Bruinsma, D. A. Hess, E. J. Mulder, B. Wiering, S. Kok, J. Woltering, B. Raap-van Sleuwen, L. Schoonderwoerd, D. Hendriks, N. van den Elzen, I. van de Laak, M. Valk, W. van der Meij, B. J. van Wely, M. J. van Hoogstraten, M. van der Sluis, I. Paulusma, M. J. W. Möllers, R. Looijen, H. C. J. van der Mijle, I. T. A. Pereboom, P. M. C. Tijink-Callenbach, R. A. Schasfoort, W. van de Meer, M. Lubberink, M. van Haskera, F. Wit, M. Jeeninga, R. ten Hoeve, F. C. W. Slootmans, B. Inberg, L. de Nes, Dianne Toonen, M. A. Wilmsen, O. Buyne, F. Ferenschild, C. Adamse, B. L. Hettema-Beets, M. K. Goudswaard, M. van der Velde, D. W. Elving, R. E. Arends-Smit, J. R. Buiter, I. van der Witte-van Aerle, K. Jansma, L. Kooistra, S. Lohof-Venema, M. R. Kruijer, G. Dijkstra, M. A. van der Werf-Elling, V. Kats-de Boer, A. M. Rinsema, M. Haarlemmer - Lutjeboer, A. Van der Vegt, S. M. H. Berends-Pors, A. J. Ponstein, G. Klaassen, A. M. Nieuwint, M. Veninga-Jansen, V. Dries-Jansen, F. J. Arends, N. E. Stellingwerf-Goinga, N. W. G. Overmars, H. Van Asma, K. Beverdam, M. J. A. C. Ploumen, M. Tijhuis, A. H. Visser Duiven, M. Former, M. A. L. Smans-Kaal, C. M. J. Vorsterman van Oijen-Linthorst, N. N. Hövels-Kamp, L. R. Vorsteveld, N. Vermeulen, A. Alkemade- van Veghel, L. J. W. Steentjes, H. G. M. Cornelisse-Theunissen, J. Strijbosch, S. Sniekers, J. M. A. Oerlemans-van Oijen, H. M. J. Hoefnagels, C. J. D. A. Sniekers, S. Biemans, Y. Bomert-Wendt, H. G. M. van Gaal, A. H. C. W. Smulders, W. Adams, J. M. Kappen, A. M. Vermeltfoort-Jansen, M. G. C. Zegger, C. Vrielink, H. M. Slotman, N. J. H. Claessens, A. W. M. Manders-de Groot, C. T. P. G. van Beuzekom- van der Vorst, M. W. C. Swinkels- Nijssen, P. van Oeveren, J. P. F. van Leeuwen-Nellestijn, M. Bleijenberg, J. J. F. Valenteyn-Hidden, M. G. van Rutten-de Groot, M. van den Nieuwenhuizen, P. G. Boorsma, N. Broodman, M. E. Elling, E. Bokkers-Engelen, G. H. Hilhorst-Droppers, H. J. C. Mein

**Affiliations:** 1 Department of Surgery, University Medical Center Groningen, Groningen, The Netherlands; 2 Department of Surgery, Radboud University Medical Center, Nijmegen, The Netherlands; 3 Isala Clinics, Zwolle, The Netherlands; 4 Department of PGSoM, University Medical Center Groningen, Groningen, The Netherlands; 5 Department of Surgery, Medical Center Leeuwarden, Leeuwarden, The Netherlands

**Keywords:** Quality of life, Fecal incontinence, Low anterior resection, Pelvic floor rehabilitation, Functional outcomes, Low anterior resection syndrome, Rectal cancer

## Abstract

**Background:**

After low anterior resection (LAR), up to 90% of patients develop anorectal dysfunction. Especially fecal incontinence has a major impact on the physical, psychological, social, and emotional functioning of the patient but also on the Dutch National Healthcare budget with more than €2000 spent per patient per year. No standardized treatment is available to help these patients. Common treatment nowadays is focused on symptom relief, consisting of lifestyle advices and pharmacotherapy with bulking agents or antidiarrheal medication. Another possibility is pelvic floor rehabilitation (PFR), which is one of the most important treatments for fecal incontinence in general, with success rates of 50–80%. No strong evidence is available for the use of PFR after LAR. This study aims to prove a beneficial effect of PFR on fecal incontinence, quality of life, and costs in rectal cancer patients after sphincter-saving surgery compared to standard treatment.

**Methods:**

The FORCE trial is a multicenter, two-armed, randomized clinical trial. All patients that underwent LAR are recruited from the participating hospitals and randomized for either standard treatment or a standardized PFR program. A total of 128 patients should be randomized. Optimal blinding is not possible. Stratification will be done in variable blocks (gender and additional radiotherapy). The primary endpoint is the Wexner incontinence score; secondary endpoints are health-related and fecal-incontinence-related QoL and cost-effectiveness. Baseline measurements take place before randomization. The primary endpoint is measured 3 months after the start of the intervention, with a 1-year follow-up for sustainability research purposes.

**Discussion:**

The results of this study may substantially improve postoperative care for patients with fecal incontinence or anorectal dysfunction after LAR. This section provides insight in the decisions that were made in the organization of this trial.

**Trial registration:**

Netherlands Trial Registration, NTR5469, registered on 03-09-2015.

Protocol FORCE trial V18, 19-09-2019.

Sponsor Radboud University Medical Center, Nijmegen.

## Background

The treatment of rectal cancer has improved greatly over recent years in terms of oncological outcomes. Combinations of surgery, radiotherapy and/or chemotherapy are responsible for improved survival data. Nowadays most of these patients can be treated with a sphincter-saving technique, such as the low anterior resection (LAR). Unfortunately, the majority of these patients develop anorectal dysfunction [1], which can consist of urgency, increased frequency of defecation, fecal incontinence, soiling, no control on flatus or incomplete evacuation. Up to 76–90% of these patients report a combination of these complaints, which are described as low anterior resection syndrome (LARS) [2, 3]. Fecal incontinence especially has a major [1, 4, 5] impact on the physical, psychological, social, and emotional functioning of the patient [3, 6].

Alongside the impact on personal life, fecal incontinence has a substantial impact on the National Healthcare budget with more than € 2000 spent per patient per year in the Netherlands [7]. Production losses in paid and unpaid work accounted for more than half of the total costs, and costs of healthcare visits accounted for almost a fifth of the total costs. One-tenth of the total cost is associated with protective material (only partially reimbursable), while incontinence medication was only responsible for 5% of the total costs [7]. Currently, the exact prevalence of LARS in the Netherlands is unknown, but an increase in the numbers of patients suffering LARS in the Netherlands is expected due to the start of colorectal cancer screening in January 2014 among men and women between 55 and 75 years.

Despite the large impact of LARS, no gold standard exists to treat these patients in a way that focuses on the cause of the problem. The current standard treatment is focused on symptom relief, consisting of pharmacotherapy with bulking agents and/or anti-diarrheal medication. However, no sustained clinical improvements of these therapies in LARS patients have been reported. Pelvic floor rehabilitation (PFR) is one of the most important treatments for fecal incontinence in general, with success rates of 50–80% [8–10]. Based on previous studies, we hypothesize that PFR might reduce the number and severity of fecal incontinence after rectal resections by 25% (measured by the Wexner score) [11–15].

The FORCE trial randomizes rectal cancer patients after sphincter-saving rectal resection for either a standardized pelvic floor rehabilitation program or standard treatment to investigate which arm results in a greater decrease of complaints and costs of fecal incontinence.

## Methods/Design

### Study design and research questions

The FORCE trial is a multicenter, two-armed, randomized controlled trial. The severity of fecal incontinence (FI) in patients after LAR will be measured by the Wexner score. Secondary study objectives are to determine the effect of PFR compared to standard treatment on the quality of life (by measuring the fecal incontinence quality of life and using the EORTC Colorectal Quality of Life Questionnaire QLQ-CR29), to analyze the cost effectiveness of full implementation of PFR compared to present daily practice (standard treatment) in treating and preventing functional bowel complaints in patients after LAR. In addition, this study aims to explore the effect of demographic, surgical and oncologic parameters on the development of FI after LAR relative to PFR and standard treatment.

### Study population

The study population consists of rectal cancer patients living in the Netherlands who undergo sphincter saving surgery (low anterior resection, LAR) in one of the participating hospitals (*n* = 17): Radboud University Medical Center Nijmegen, University Medical Center Groningen (UMCG), Medical Center Leeuwarden (MCL), Isala Clinics Zwolle, Antonius Hospital Sneek, Nij Smellinghe Drachten, Ommelander Hospital Groningen (OZG), Canisius-Wilhelmina Hospital (CWZ) Nijmegen, Deventer Hospital, Slingeland Hospital Doetinchem, Máxima MC (MMC) Veldhoven/Eindhoven, Treant Zorggroep Emmen, Bernhoven Uden, Tjongerschans Heerenveen, Elizabeth TweeSteden Ziekenhuis (ETZ) Tilburg, Maasziekenhuis Pantein Boxmeer, and Streekziekenhuis Koningin Beatrix (SKB) Winterswijk. In each center, a principal investigator is assigned for inclusion and informed consent (Additional file 1). In case of a temporary stoma, patients can participate until 6 weeks after closure. Without a temporary stoma, patients can participate 3 months after closure. Inclusion criteria are (1) adults (≥18 years), (2) LAR for rectal carcinoma, and (3) being intellectually and/or linguistically capable of understanding the questionnaires. Patients with a history of proctitis, colitis ulcerosa, or Crohn’s disease; a life expectancy of less than 1 year; and mental or physical inabilities to undergo PFR are excluded. Participants with locally advanced (T4) tumors indicated for extensive resection (beyond TME) and patients who received invasive physiotherapy during the previous 6 months are excluded as well.

### Flow of the FORCE trial

**Fig. 1:**
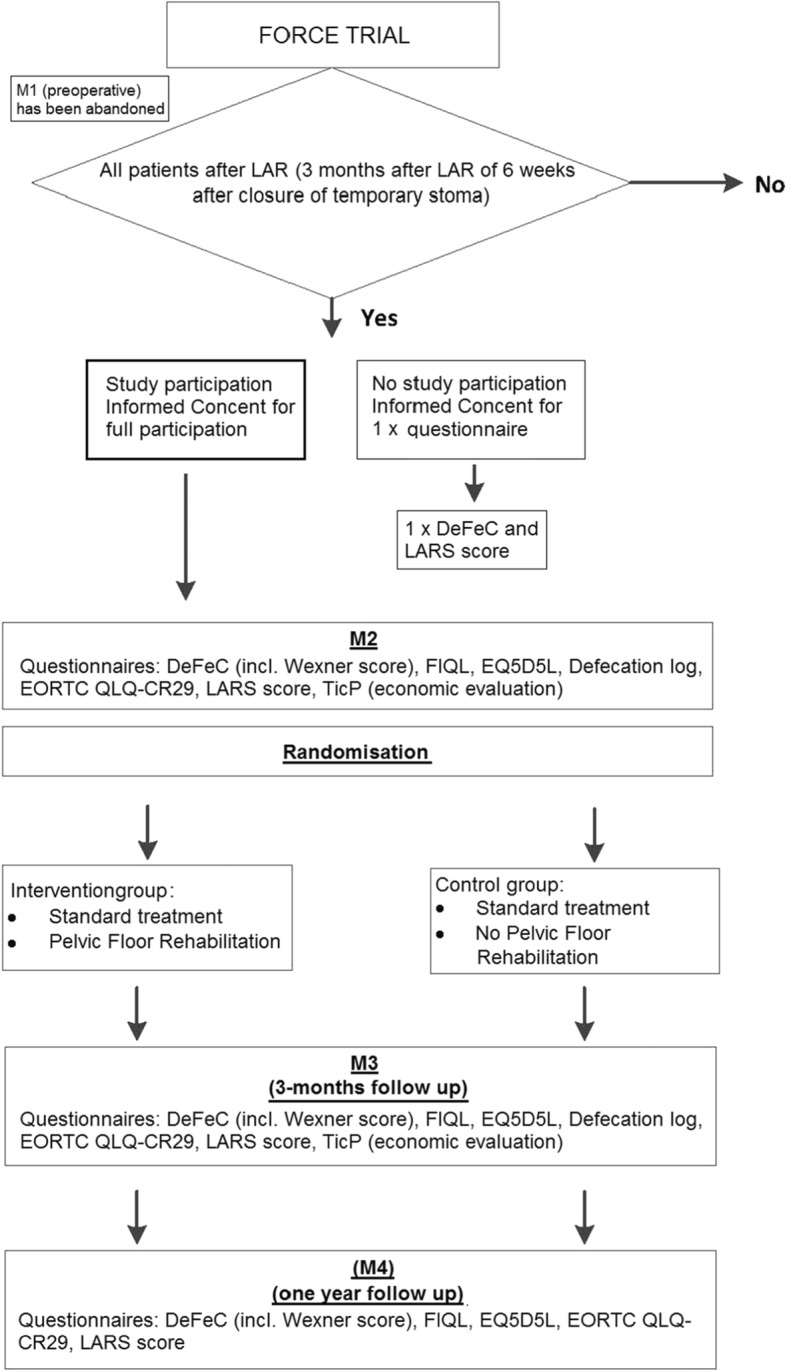
Flowchart of the FORCE trial. Abbreviations: M1–4, measurement 1 till 4. LAR, low anterior resection

After surgery, eligible patients will be randomized for either a standardized pelvic floor rehabilitation (PFR) program or standard treatment, which is defined as current daily practice. Blinding for PFR is not possible. Randomization and data collection will be performed by the coordinating investigator. In concordance with the study flowchart (Fig. 1), these steps will be followed:

1. After giving informed consent, eligible patients will be included in the FORCE trial. In the beginning of the inclusion period, this inclusion was supposed to occur preoperatively with a baseline measurement before surgery as well (measurement 1, M1). Since this preoperative inclusion led to reduced numbers of inclusion due to an overload of information, postoperative inclusion was allowed as well.
2. Questionnaires will be administered 3 months after LAR (measurement 2, M2) to patients without a temporary stoma. Based on the current reports, approximately 70% of patients undergoing LAR also receive a temporary stoma [16]. In these cases, the questionnaires will be administered 6 weeks after stoma closure (measurement 2, M2).
3. Randomization will take place after completing the M2 questionnaires. Patients will then undergo either standard treatment or a standardized PFR program that includes the standard treatment for 3 months (12 sessions, once a week).
4. After finishing the PFR program or 12 weeks of participation in the control group, questionnaires will be send again (measurement 3, M3, primary endpoint).
5. Long-term follow-up will be completed at 1 year after bowel continuity (measurement 4, M4).

Patients who decline the request for participation will be asked to fill out the DeFeC questionnaire (with Wexner score included) and LARS score only one time. These patients will sign a separate informed consent form for this step (Additional file 2). Study participation for these patients will stop directly after finishing these questionnaires. By means of this questionnaire, a valid statement can be made about the potential selection bias for participation that is based on the amount of complaints.

Patients from the control group who would like to undergo PFR will be given the opportunity to undergo this treatment after finishing measurement 3 (M3). These patients will undergo an identical PFR program as the patients that are initially randomized to this intervention and will be asked to fill in another questionnaire (similar to M2/M3) after their treatment. This might provide useful information about whether the timing of PFR is of influence on the results.

This study protocol is in accordance with the 2013 Standard Protocol Items: Recommendations for Interventional Trials (SPIRIT) Statement [17]. The checklist can be found in Additional file 3. The schedule of enrollment, interventions, and assessments is shown in Table 1.

**Table 1:** The schedule of enrollment, interventions, and assessments (according to the SPIRIT statement 2013)

TIME POINT		STUDY PERIOD				
	Enrollment	Allocation^a^	Post-allocation			Close-out
	October 2017 – December 2019	0	t_1_^b^	t_2_^b^	t_3_^b^	December 2019 – June 2020.
ENROLLMENT:						
Eligibility screen	X					
Informed consent	X					
Randomization and allocation		X				
INTERVENTIONS:						
Pelvic Floor Rehabilitation			X			
Control group			X			
ASSESSMENTS:						
Questionnaires to assess study outcomes			X	X	X	
Statistical analysis						X

^a^Allocation of the intervention/control group takes place 3 months after index surgery without temporary stoma construction or 6 weeks after stoma reversal if initial surgery included stoma construction. Measurement t1 will always take place before randomization/allocation

^b^*t1* measurement before randomization, *t2* primary endpoint (12 weeks after control/intervention period), *t3* one year follow-up

### Recruitment and informed consent

The Committee on Research Involving Human Subjects Arnhem-Nijmegen approved the FORCE trial. All patients with proven colorectal cancer will be treated according to standard surgical protocols. Patients who meet the inclusion/exclusion criteria will be selected and asked for participation for this study by their own surgeon. The surgeon will inform the patients by handing out the patient information and will ask for permission to inform the coordinating investigator about his/her possible participation. This step will be noted in the electronic health records. After a reasonable time (2 weeks), the coordinating researcher will call the patient to answer potential questions. The patients will have 2 weeks to consider before renewed contact is made. When patients decide to participate, they will sign the Informed Consent and send it to the coordinating investigator at Radboud University Medical Center (Radboudumc). The coordinating investigator will be responsible for storing the signed Informed Consent in the Trial Master File at Radboudumc and in the investigator site files at the participating centers. On the consent form, participants will be asked if they agree to the use of their data. Participants will also be asked for permission for the research team to share data with the regulatory authorities, where relevant.

### Randomization, stratification, and blinding

#### Randomization and stratification

Randomization will be performed 3 months after the LAR in patients without stoma construction, and in case of a temporary stoma, 6 weeks after stoma closure. The coordinating investigator will conduct the randomization procedure, using Castor EDC (www.castoredc.com). Stratification will be done in variable blocks. It will be stratified for gender and radiation because of frequent sphincter function impairment due to birth trauma in women and the association between radiation and sphincter and bowel dysfunction. The coordinating investigator will be responsible for communication with the pelvic floor specialized physiotherapists and will inform them about referral and the allocated intervention.

#### Blinding

The physiotherapists will be informed about the pre- and postoperative medical history of the patient. Surgeons and physiotherapists are blinded in terms of outcomes of questionnaires taken before the start of PFR (measurement M2, Fig. 1). Complete blinding of the allocated intervention for patients and the participating physiotherapists is impossible. Unblinding will not occur, since the design is open label. The coordinating investigator, who will also be involved in data analysis, is not blinded for the allocation since he will inform the physiotherapists about referral and the allocated intervention. Additional data analysists will be blinded for allocation.

### Data analysis and statistics

#### Primary outcome

The primary outcome is based on the Wexner score. The dependent variable is the severity of FI measured by this score. The independent variable is the intervention or the standard treatment. The difference between the intervention and control group will be analyzed by an analysis of covariance (ANCOVA), with the baseline measurement as covariate. This test is preferred over the T-test since this analysis reduces the within-group error variance. Therefore, the precision of the treatment estimate is increased, and the length of the confidence interval is reduced [18]. The M3 measurement will be valid as the primary outcome. The M4 measurement, which is used to assess sustainability of results, will be influenced by the patients of the control group who will choose to undergo PFR after finishing their primary endpoint measurements (after 3 months of standard treatment, i.e., in case of severe FI). For statistical analysis, the effect between the intervention group and control group will be evaluated with and without this group to measure the influence of this group on the M4 measurement. The same analysis of covariance (ANCOVA) will be used.

#### Secondary endpoints

- The economic effects of full implementation of PFR compared to standard treatment in treating and preventing FI in patients after LAR will be determined by a cost effectiveness analysis. This will be evaluated by regression analysis (*see* subheading “cost effectiveness analysis” below for detailed methods).
- The effect of the demographic, surgical, and oncologic parameters on the development of FI after LAR relative to PFR and standard treatment will be explored.
- The effect of PFR compared to standard treatment on anorectal outcomes in patients after LAR by the DeFec, LARS score, and defecation log will be determined.
- The effect of PFR compared to standard treatment on QoL by means of the Fecal Incontinence Quality of Life (FIQL), EORTC Colorectal Quality of Life Questionnaire QLQ-CR29, and EQ5D will be determined. The latter evaluation will be used for economic evaluations in particular.

The dependent variable is the degree of QoL/anorectal outcome. The independent variable is the intervention or the standard treatment. The difference between the effect in the intervention group and the control group will be analyzed by analysis of covariance (ANCOVA) as well. No additional analyses, such as subgroup analysis, are written out in advance.

#### Intention to treat and per protocol analysis

Primary study outcome will be based on intention to treat analysis. In addition, per protocol analysis will be performed. This analysis will be done to avoid the effects of crossover, protocol violation, and dropout.

#### Handling and storage of data and documents

The anonymized data will be stored in Castor EDC. Only the coordinating and principal investigators will have access to the key of the code that matches participants to study data. Most patients will fill in their questionnaires themselves online. Only those without email will fill in the paper version of the questionnaires.

No specific plans, besides an active attitude towards the participating centrums, are made to promote participant retention and complete follow-up. If patients fail to start/continue their allocated intervention (Pelvic Floor Rehabilitation, since failure of control group is not possible), data will be collected according to the study protocol for use in per protocol analysis.

### Sample size calculation

Recent studies reported an improvement of FI after LAR by PFR of five points on the Wexner-score (SD = 8) [11, 12, 15]. This improvement is considered to be of great clinical significance.

In the original design of this trial, an independent T-test was suggested for the primary outcome measure. Progressive insights made it possible to make a change to the ANCOVA analysis, since this test reduces the within-group error variance. Therefore, the precision of the treatment estimate has been increased, and the length of the confidence interval has been reduced [18]. Therefore, for this trial with an ANCOVA as primary statistical test, the following sample size calculation was made:

1. The T-test sample size was based on 80% power to detect a difference of five points in the Wexner score, SD = 8, two-grouped t-test, with a 0.05 two-side significance level: 63 patients per arm.
2. The sample size calculation for analysis of covariance (ANCOVA) in randomized clinical trials described by Borm et al. [18] was used, with an estimated correlation factor (R) of 0.5. The required sample size is 32 patients per arm.
3. Assuming a withdrawal/replacement rate of 50% [11, 12, 15], a total of 128 patients should be randomized.

### Data monitoring, harms, and auditing

The Committee on Research Involving Human Subjects, Arnhem-Nijmegen approved this study and declared it as a “negligible risk” study. Therefore, no Data Safety Monitoring Board is needed, and no interim analysis or formal stopping rules for the trial are needed to be conducted or formulated. No anticipated harms exist, nor will compensation be provided for trial participation. No need for post-trial care is expected. Monitoring will occur with a frequency of one visit per year per center, in which the following items will be checked: informed consents, availability of data in Trial Master File and Investigator Site Files, inclusion and exclusion criteria, SAEs, and source data verification. Trial auditing will be twice a year since that is the frequency of meeting with the Trial Steering Group of principal investigators. Any solicited and spontaneously reported adverse events and other unintended effects of the trial will be reported to the Committee on Research Involving Human Subjects, Arnhem-Nijmegen, who will review the situation and give appropriate advice regarding expectedness, seriousness, severity, and causality.

### Patient information and questionnaires

#### Medical history/patients characteristics

The patient information will include the medical history, gender, age, tumor height (MRI and scoping), height and type of the anastomosis, type of surgery (laparoscopic, open or robotic), construction of a temporary stoma, peri- and postoperative complications, time of surgery, blood loss, time of hospitalization, and clinical and pathological TNM staging.

#### Groningen defecation and fecal continence (DeFeC)

This questionnaire incorporated various Rome IV criteria and scoring tools for severity of constipation and fecal incontinence. Overall reproducibility of the Groningen DeFeC questionnaire is acceptable and its validity is good [19]. This makes it a feasible screening tool for defecation disorders. The primary outcome, the Wexner score, will be derived from this questionnaire. The distribution of subtypes and symptoms of fecal incontinence in the general Dutch population has already been investigated with the use of this questionnaire [20].

#### Wexner incontinence score

The Wexner score allows the severity of fecal incontinence to be assessed using five questions (Table 2). The minimal score patients can obtain is 0 (continent), and the maximum score is 20 (highest severity of incontinence). This score describes the type and frequency of the incontinence and impact on daily life. No data exist about the internal consistency and the criteria/content validity of this score. The intraclass correlation coefficient (ICC) of the Wexner score is good to excellent (ICC 0.75). The construct validity is R(Pearson) = 0.78 (correlation with Vaizey score) [21]. The Wexner score has an important association with the Fecal Incontinence Quality of Life score (r = − 0.45), but a weak correlation with changes in the EQ-5D has been described [22].

**Table 2:** Wexner incontinence score

Type of incontinence	Frequency				
	Never	Rarely	Sometimes	Usually	Always
Solid	0	1	2	3	4
Liquid	0	1	2	3	4
Gas	0	1	2	3	4
Wears pad	0	1	2	3	4
Lifestyle alteration	0	1	2	3	4

Never, 0; rarely, < 1/month; sometimes, < 1/week, ≥1/month; usually, < 1/day, ≥1/week; always, ≥1/day

0, perfect; 20, complete incontinence

#### LARS score

The LARS score is a validated scoring system for bowel dysfunction after low anterior resection for colorectal cancer. The Dutch version was recently validated [23]. This score covers the five most bothersome issues in terms of prevalence and impact of QoL, namely incontinence for flatus and liquid stool, frequency, clustering, and urgency. Scores range from 0 to 42, with subdivisions in three categories: no (0–20), minor (21–29), and major LARS (30–42). A statistically significant association exists between a higher LARS score and an impaired QoL. The test-retest reliability of the LARS score is good, with an interclass correlation coefficient of 0.79 [23].

#### Fecal incontinence quality of life score (FIQL score)

The FIQL score is a condition-specific questionnaire on quality of life and consists of four multi-item subscales: lifestyle (10 items), coping/behavior (nine items), depression/self-perception (seven items), and embarrassment (three items), for a total of 29 items [24]. Each item has four answer opportunities, with a score of 1 to 4, where 1 indicates a low quality of life and 4 a high quality of life.

The internal consistency (Cronbach’s α) is good (subscale 1, 0.96; subscale 2, 0.96; subscale 3, 0.88; and subscale 4, 0.80). The total FIQL has an adequate internal and external responsiveness (standardized response mean = 0.5, r = − 0.48, and area under the curve = 0.765). The intraclass correlation coefficient is good to excellent: 0.80 (embarrassment) to 0.93 (lifestyle) [25].

#### EORTC colorectal quality of life questionnaire QLQ-CR29

Quality of life will be measured by the EORTC Colorectal Quality of Life Questionnaire QLQ-CR29. The ICC of the QLQ-CR29 is good to excellent (Intraclass Correlation Coefficient: 0.78) [26, 27]. The Internal consistency of this questionnaire reaches the criterion of 0.70 [26].

#### Defecation and urinary log

This log provides insight into the defecation pattern, type of consistency (Bristol Stool Form Scale) [28], feeling of urge to defecate, episodes of fecal incontinence, and the use of diapers. Also, the use of medication will be noted. The patient will keep up this log 5 days per week after LAR or temporary stoma from the moment of randomization until the end of the intervention (3 months). This log will be used to allow discussion about the number of treatment sessions that will be needed to obtain an effect of PFR. Fecal incontinence occurs often in combination with incontinence for urine. In case of urine incontinence, the patient has to keep up the severity and episodes of this urinary incontinence. The incontinence will namely influence the costs (i.e., use of diapers).

### Cost effectiveness analysis (CEA)

The economic evaluation investigates, alongside the clinical trial, the value for money of full implementation of the standardized pelvic floor rehabilitation program compared to the usual care. This will be done from a societal perspective. The timeframe of empirical evaluation is 6 months. The effect at 6 months is supposed to be sustainable for 5 years, which will be explored by decision-analytic modelling. The design of the economic evaluation follows the principles of a cost-utility analysis and adheres to the new Dutch guideline for performing economic evaluations in health care (ZINL, 2015). Fecal incontinence-related problems are anticipated to decrease after PFR, which would result in a reduction of health care consumption, increased work resumption/participation, and an increase in health-related quality of life in this target population. Cost-effectiveness will be expressed in terms of gains in the cost per Quality Adjusted Life Year (QALY). Uncertainty will be dealt with by one-way sensitivity analysis (deterministic) and by parametric statistics, ultimately presenting cost-effectiveness acceptability curves. The modelling part will be probabilistic.

#### Cost analysis

The cost analysis exists of two main parts. First, at the patient level, the volume of care will be measured prospectively over the time path of the study using patient-level diaries (on fecal incontinence-related health care consumption), completed, if necessary, by data from the patient training facility’s administration system. The diary will be developed in a way that it structures and makes uniform the health care consumption of a fecal incontinent patient for this particular target population. Second, per item of health care consumption, the standard cost will be determined using the guideline for performing economic evaluations (ZINL, 2015), which will be completed with the total costs of items via activity-based costing. Productivity losses for patients will be assessed using a patient-based diary complemented with an interview on a 3-month recall basis between the researcher and the patient (at baseline and at the 3- and 6-month follow-ups). The friction cost-method will be applied following the Dutch guidelines (ZINL, 2015). In addition, travel time to the training site and related costs will be considered (also on the basis of the diary). Differences in costs between both groups will be evaluated using regression-based techniques.

#### Patient outcome analysis

The effect analysis adheres to the design of the randomized controlled trial and measures at baseline and at fixed points along time path/follow-up of the clinical trial (see design clinical trial). For measurements on the quality of the health status of the patients, a validated so-called health-related quality of life (HR-QoL) instrument—the EuroQol-5D (EQ-5D) [29]—will be used. This HR-QoL instrument will be completed by the patients and is available in a validated Dutch translation. The EQ-5D-5 L which is used in this study is a generic HR-QoL instrument comprising five domains: mobility, self-care, usual activities, pain/discomfort, and anxiety/depression [30, 31]. The EQ-5D-5 L index is obtained by applying predetermined weights to the five domains. This index gives a societal-based global quantification of the patient’s health status on a scale ranging from 0 (death) to 1 (perfect health). Patients will also be asked to rate their overall HR-QoL on a visual analog scale (EQ- 5D VAS) consisting of a vertical line ranging from 0 (worst imaginable health status) to 100 (best imaginable). For the EQ-5D-3 L, the version used before the introduction of the EQ-5D-5 L, a weak correlation was known to exist with changes on the Wexner score [22]. The same can be expected from the EQ-5D-5 L.

#### Budget impact analysis (BIA)

BIA will be conducted to assess how healthcare budgets will be influenced when offering the standardized pelvic floor rehabilitation program. This program is anticipated to allow savings of €191 in direct medical cost and €280 in productivity gains for the average patient on a yearly basis. The patient-based budget impact will be extrapolated to the population level. Although the BIA relies heavily on the findings from the economic evaluation described above, it will be conducted from various perspectives, such as from the broader perspective of the public purse down to the narrow healthcare perspective. To that end, use will be made of a health economic (decision analytical) model in which uncertainty will be taken into account. Deterministic uncertainty concerning BIA input, such as perspective, pricing parameters, time horizon, uptake, etc., will be dealt with by generating the budget impact as a series of sensitivity analyses covering a relevant range of costs. In general, for the BIA we adhere to the new guideline for performing economic evaluations in health care (ZINL, 2015).

### Investigational treatment

#### The control group

Patients in the control group will get the standard treatment that currently is used in daily practice. This standard treatment for postoperative FI consists of the prescription of bulking agents (i.e., Metamucil, psyllium fibers, Volcolon, or Normacol). Bulking agents can be beneficial to the consistency of the stool and therefore diminish soiling problems. These bulking agents may be used once or twice per day depending on the severity of FI. Standard oncologic follow-up after LAR at the surgical outpatient clinic will be provided. In case of severe FI and/or failing of standard treatment, cross over to the intervention group will be allowed. After finishing their allocated control group period, these patients with severe complaints might wish to undergo PFR. This will be allowed, and these patients will receive PFR according to the study protocol. After 12 sessions, patients will be asked to fill in another questionnaire, which will allow them to evaluate whether the length of the post-operative period after which PFR has been applied, influenced the patient’s outcomes.

#### The intervention group

Treatment of patients in the intervention group will consist of four modalities of pelvic floor rehabilitation in addition to the standard treatment:

1) pelvic floor muscle training to a) increase the maximum strength of muscle contraction, b) extend the time of muscle contraction, and c) improve the coordination of contraction of the pelvic floor muscles; 2) biofeedback, which is a behavioristic therapy that allows the patient to become conscious of the contraction and relaxation of the pelvic floor muscles and uses an anal electromyography probe; 3) electrostimulation, which can improve the effectiveness of the contraction strength of the pelvic floor muscles and uses the same anal electromyography probe as used for biofeedback; and 4) training with a rectal balloon to simulate the urgency to defecate, which is a method that enables the patients to train to retain stool, enabling the patient to retain a larger stool in the rectum.

The intervention will be performed by specialized pelvic floor physiotherapists, registered in the Dutch KNGF-NVFB register. These physiotherapists will be chosen based on the location of their practices. For the comfort of patients included in this study, the close location of the practices to the participating hospitals is important. Therefore both hospital-based and private practices were allowed to participate. The physiotherapists will be obliged to attend a course during which the treatment protocol and the case report form will be explained. The coordinating investigator will guide this course and will train the physiotherapists. During this training session, the correct way of digital rectal examination to assess the function of the pelvic floor, the use of biofeedback, electrostimulation, and the rectal balloon will be explained and illustrated using videos. An internationally accepted protocol, The Pelvic Floor Assessment Protocol, will be used for the digital rectal examination performed by the physiotherapist (International Continence Society 2006, Messelink et al.). The physiotherapist will also be instructed on how to use the case report form and report any adverse events during the 12 sessions of PFR. Since a patient’s adherence to PFR is a common problem, an instruction on how to enlarge this adherence to therapy will be provided [21, 22]. The complete treatment trajectory for both the control and the intervention group will be recorded in a standardized protocol, which has already been developed by clinical experts in the field [8].

#### Pelvic floor muscle training

Patients will start with the intervention trajectory 3 months after LAR or 6 weeks after stoma closure. During the next 3 months, they will undergo 12 treatment sessions, once per week. The first session will take 45 min, and the following sessions 30–35 min. During every treatment, the physiotherapist will complete a case report form. This case report form contains all information concerning pelvic floor muscle training, biofeedback, electrostimulation, peri-anal examination, and digital rectal examination. Digital rectal examination of pelvic floor functionality will be used to assess the ability to consciously contract the pelvic floor muscles and to quantify the strength of the contraction [8, 32]. The use of medication according the standard treatment will be reported also in the case report form. All patients randomized to the intervention group will be instructed by their physiotherapist on how to exercise the pelvic floor. The patients will be thought to selectively generate voluntary contractions of the puborectal muscle and the external anal sphincter and also how to relax these muscles and to avoid co-contractions of other muscles. The pelvic floor has to satisfy requirements of maximal strength, progression of the duration of the strength, and progression in timing and coordination of the contraction [8, 32]. Additionally, folders will be provided, with illustrations and short descriptions of exercises, which have to be performed at home three times per day, preferably at fixed time points [8, 32]. The success of PFR depends greatly on the motivation, willingness, and self-discipline of patients to exercise at home [8, 32].

##### Biofeedback

Biofeedback is a cognitive behavioral therapeutic intervention, which is used during PFR exercises to help patients in monitoring their pelvic floor function. Biofeedback provides insight in the activity of the pelvic floor and gives the patient direct feedback during exercises [32]. Biofeedback will, if available at the local PFR clinic, be achieved by an anal electromyography (EMG) probe, with 24 sensory points, located at six different heights and four different directions along the probe. The MAPLe system is validated for its purpose and the selected physiotherapists are already experienced users (MAPLe, Novuqare, the Netherlands) [24]. If this MAPLe system is not available in the participating PFR clinic, use of the Anuprobe anal probe (Pelvitec, the Netherlands) is acceptable to perform the described intervention. Biofeedback will be conducted during all PFR sessions.

##### Electrostimulation

Electrostimulation will be used to gain strength and effectiveness of contractions of the pelvic floor. Especially when contractions of the pelvic floor muscles are not observable or palpable, electrostimulation is able to train the pelvic floor muscles and will contribute to better strength of contraction [32]. Electrostimulation will be conducted during all PFR sessions in combination with biofeedback using the same anal probe.

##### Rectal balloon training

Rectal balloon training is used to simulate the need for defecation. During training, a rectal balloon (Ashley Rectal Balloon, Pelvitec) will be inserted into the neorectum (i.e., remaining part of rectum and distal colon). Subsequently, the balloon will slowly be inflated using a syringe, which is connected to the balloon. Patients will be asked to pay attention to their rectal filling sensations and once they feel a strong desire for defecation, an adequate contraction of the sphincter and puborectal muscle needs to be produced to retain the balloon. The awareness that also their neorectum can adapt fecal content and that therefore, after a short period of active sphincter/pelvic contraction, the urge for defecation will diminish will strengthen their confidence in fecal continence [32]. Rectal balloon training helps to control fear of FI and allows patients to tolerate larger volumes of stool in the neorectum. Rectal balloon training will not be started until the patients’ maximum pelvic floor functions is recovered. During the last three PFR sessions, this training will be part of the sessions anyway. Rectal balloon training will be combined with biofeedback.

### Use of cointerventions

In a selected case of severe FI, colon irrigation or permanent colostomy might be valuable alternatives when the standard treatment does not decrease either the severity of FI or diarrhea. Such procedures will be considered cointerventions. Diarrhea is frequently seen after LAR and might contribute to the severity of FI; therefore, diarrhea inhibitors (usually loperamide derivates) are often prescribed. When this medication is already being used on a regular basis at the start of the intervention, patients will be instructed to continue this during the study. Cointerventions or any changes in medication will be reported in the case report form by the pelvic floor physiotherapist or the surgeon. This information will be taken into consideration during statistical analyses.

## Discussion

To our knowledge, the FORCE trial is the first to study the effects of a structured pelvic floor rehabilitation (PFR) program after sphincter-saving rectal cancer surgery in a prospective randomized controlled trial with a well-defined rehabilitation program that uses all four important parts of pelvic floor training (pelvic floor muscle training, biofeedback, electrostimulation, and rectal balloon training) and includes an evaluation of quality of life and cost-effectiveness.

Previous studies that evaluated PFR after low anterior resection differed in terms of patient selection criteria and study design but differed most of all in the PFR protocols and fecal incontinence scoring systems [11–15] used. Visser et al. (2014) noted the importance of consistent quality of life assessment in future trials with PFR since only one study had assessed this outcome before [33]. This study protocol meets this need and will add a cost-effectiveness analysis for full implementation of the PFR.

The authors choose to publish this study protocol to prevent wasteful duplication of research effort and expenses but most of all to provide an insight into the decisions that were made during designing the FORCE trial. Several points will be discussed below.

### Choices of questionnaires

The outcomes of this study will be assessed by standardized questionnaires. To date no consensus exists on how to evaluate the severity of fecal incontinence and its consequences for the Quality of Life [21, 22, 34, 35]. A combination of the Wexner score and FIQL score is considered to give the most objective outcomes [21], which is why these were incorporated into the study design. The DeFeC is used since this questionnaire incorporates various Rome IV criteria and scoring tools for constipation and fecal incontinence but also has reference population data available, which is important for the interpretation of results [20]. For a better insight into the quality of life after LAR, the EORTC QLQ-CR29 was chosen. This list was preferred over several other questionnaires (i.e., the RAND36 or WHOQoL) since this one is a colorectal-specific module while the others asses the QoL from a general point of view. The presence of comorbidity, unrelated to defecation problems, in this aging group of patients might otherwise interfere with the reported quality of life.

The choices for the other questionnaires do not require additional explanation in our opinion.

### Inclusion of patients regardless of the degree of their complaints

Where several previous studies selected patients with a specific amount of complaints/incontinence, we choose to include all patients without a predefined selection on the degree of postoperative complaints. This decision was based on the high percentage of patients that experience different types of functional bowel complaints after LAR and the hypothesis that all patients receiving the operation possibly could benefit from PFR. In addition, no uniformity exists in the cut-off value for our chosen primary endpoint, namely the Wexner incontinence score. The additional advantage of including unselected patients is that it allows a valid and more extensive statement to be made about a broad spectrum of patient’s characteristics, including their continence status, which are important in predicting a specific outcome after PFR.

### Postoperative inclusion

The FORCE trial protocol was initially based on preoperative inclusion and included a preoperative measurement. Unfortunately, patients experienced an overload of preoperative information, and they stated that they wished to focus on their planned surgery. Therefore, we encountered serious difficulties in enrolling patients in the study, which is why we chose postoperative inclusion only. We are aware that this decision might introduce a selection bias, since patients already know the severity of their complaints. To report which specific type of patients refuse to participate, we started to ask patients who had denied full study participation to only fill in a single questionnaire regarding functional bowel complaints. We are also aware of the difficulties in reporting valid statements on the patient’s continence level before surgery. Determining the impact of surgery and/or radiotherapy in consideration of the preoperative continence level of the patient would be interesting, but this turned out to be infeasible. Since patients with serious postoperative complications (i.e., anastomotic leakage) are in a poor condition after surgery, this might induce selection bias as well. Therefore, this study likely cannot provide hard conclusions on FI and PFR after such serious problems.

### Extensive anorectal manometric and function testing

Having patients fully examined with regard to manometry and anorectal functioning and objectively measuring what the effects of PFR are after rectal surgery [36] would have been interesting. Especially since rectal (cancer) surgery with the construction of a low anastomosis might interfere with the puborectal continence reflex [37, 38] and could induce clinical symptoms of fecal incontinence. However, such an examination turned out to be logistically infeasible for all patients due to the geographical spread of hospitals throughout the country and limited accessibility of anorectal function centers. In addition, the anorectal function devices that are currently used show a lot of variation regarding quality of measurements, which would make it difficult to make valid comparisons.

As with many randomized controlled trials, the FORCE trial is designed to make a difference in clinical daily practice. We believe that the results of this study can substantially improve care for patients with bowel dysfunction after LAR for rectal cancer. Subsequently, positive study results may be used in future guidelines, clinical practice algorithms, and eventually in the decision-making process of health insurances to reimburse PFR after LAR as standard care.

In case this study does not shows the hypothesized results, or in an insufficient amount, our considerations are in line with the statements of Bols et al. [8] that the received physiotherapeutic interventions can still be of value. The patients’ awareness and capability to coordinate their pelvic floor system have increased, which are positive and expected to be useful in the future for this aging population.

## Trial status

This is protocol version number 18 (approved 19 September 2019) of an ongoing trial. Recruitment started October 2017 and is expected to finish January 2020. Patient recruitment has not been completed at the time of submission of this article.

## Supplementary information

**Additional file 1:.** Model informed consent form, full participation (translated into English)

**Additional file 2:.** Model informed consent form, single time questionnaires

**Additional file 3:.** Standard Protocol Items: Recommendations for Interventional Trials (SPIRIT) Statement, 2013, Checklist:

## Data Availability

Not applicable now. All data generated during this study will be handled following the principles of FAIR-data (Findable, Accessible, Interoperable, and Reusable) at the end of the study.

## Abbreviations

EMG: Electromyography
FI: Fecal incontinence
FIQL: Fecal incontinence quality of life
IC: Informed consent
LAR: Low anterior resection
LARS: Low anterior resection syndrome
PFR: Pelvic floor rehabilitation
Sponsor: The sponsor is the party that commissions the organization or performance of the research, for example a pharmaceutical company, academic hospital, scientific organization or investigator. A party that provides funding for a study but does not commission it is not regarded as the sponsor but is referred to as a subsidizing party.
QoL: Quality of life
WMO: Medical Research Involving Human Subjects Act (in Dutch: Wet Medisch-wetenschappelijk Onderzoek met Mensen)
ZonMw: The Netherlands Organization for Health Research and Development
