# Supplementary material for: Pelvic floor rehabilitation to improve functional outcome and quality of life after surgery for rectal cancer: study protocol for a randomized controlled trial (FORCE trial)
Source: Trials. 2020 Jan 28;21:112. doi: 10.1186/s13063-019-4043-7 (PMC6988240; doi:10.1186/s13063-019-4043-7)
Supplement: Supplementary file 2 — Additional file 2:. Model informed consent form, single time questionnaires [file 13063_2019_4043_MOESM2_ESM.docx]

**File 2. Model informed consent form, single time questionnaires** (translated in English)

Official research title: Pelvic Floor Rehabilitation versus control group to improve functional Outcome and quality of life after surgery for Rectal CancEr: a randomized controlled trial (FORCE trial). I have been asked to give permission for participation to this scientific research:

- I have read the information letter carefully. I was in the opportunity to ask questions. My questions are correctly answered. **I had enough time to decide that I don’t want to participate completely to this study.**
- The researchers asked me to fill in a single questionnaire about my bowel function and that my part for this study stops after this single questionnaire.
- I know that participation is voluntarily. I know that I can decide to withdraw from participation at any moment. I don’t need to give any reason for this.
- I do not grant permission to inform my general practitioner/specialist and pharmacy that I participate in this study, since I only participate in a single questionnaire.
- I do not grant permission for requesting information about my medical history and medication use from my general practitioner/specialist. This is not necessary for this single questionnaire.
- I know that some people can look into my records. These people are noted in this information letter.
- I grant permission to store my data for 15 years after this trial at this research location.
- **I want to participate to a single time questionnaire about my bowel function. I do not want to participate to the full research proposal as described in this patient information.**

Name subject:

Signature: Date : __ / __ / __

----------------------------------------------------------------------------------------------------------------

I declare that I have fully informed this subject about the mentioned study. If, during this study, new information becomes available that could influence the consent of the subject, I will notify him or her in due times.

Name researcher (or representative)

Signature: Date: __ / __ / __
